# Transient regional osteoporosis of the hip with extensive bone marrow edema (BME): Dramatic improvement after three months of Alendronate therapy

**DOI:** 10.1016/j.radcr.2021.05.066

**Published:** 2021-07-03

**Authors:** Yasser Emad, Yasser Ragab, Mariam Ahmed Saad, Johannes J. Rasker

**Affiliations:** a Rheumatology Department, Faculty of Medicine, Cairo University, Kasr Alainy St, 11562 Cairo, Egypt; b Rheumatology Department, Dr. Erfan and Bagedo General Hospital, King Fahad St. (Al Sitteen), 21452, Jeddah, Saudi Arabia; c Radiology Department , Dr. Erfan and Bagedo General Hospital , King Fahad St. (Al Sitteen), 21452, Jeddah, Saudi Arabia; d Radiology Department, Faculty of Medicine, Cairo University, Kasr Al-Ainy St, 11562 Cairo, Egypt; e Medical Oncology Department, National Cancer Institute, Cairo, Egypt; f Faculty of Behavioral, Management and Social Sciences, Department Psychology, Health and Technology, University of Twente, Drienerlolaan 5, 7522NB Enschede, the Netherlands

**Keywords:** Transient regional osteoporosis of the hip, Transient bone marrow edema syndrome, Alendronate, BME, Bone marrow edema, MRI, magnetic resonance imaging, STIR, short tau inversion recovery, TOH, transient osteoporosis of the hip, AVN, avascular necrosis, TBME, transient Bone marrow edema, RSD, reflex sympathetic dystrophy, BMES, Bone marrow edema syndromes, RMO, regional migratory osteoporosis, FAI, femoro-acetabular impingement

## Abstract

Transient osteoporosis of the hip, also termed transient bone marrow edema, is a painful condition often occurring after trivial trauma. It can be diagnosed with MRI in patients whose radiographs are negative or inconclusive. In this case report we describe a 39-year-old female patient with this rare entity, who was successfully treated with oral Alendronate, active vitamin D and calcium supplementation combined with avoiding of weight bearing on the affected hip. She improved clinically within three months and on contrast enhanced MRI studies, as performed before and after treatment, complete regression of bone marrow edema was shown already after three months of treatment. The literature was reviewed regarding the pathophysiology of transient osteoporosis of the hip and the beneficial effects of Alendronate in this domain. The report is important because it will increase the awareness among clinicians and radiologists about this entity, as in neglected cases transient regional osteoporosis of the hip may progress to avascular necrosis with complete loss of hip function.

## Introduction

Bone marrow edema (BME) is a radiological term describing a region with low signal intensity on *T1*-weighted magnetic resonance imaging (MRI) but with high signal intensity on *T2* MRI or in short tau inversion recovery sequences [1,2]. BME affecting the hip joint(s) is neither a specific MRI finding nor a specific diagnosis [1]. In the hip joint, BME can be encountered in diverse hip disorders in both adult and pediatric populations with different underlying etiologies like inflammatory arthropathy, transient osteoporosis of the hip (TOH), avascular necrosis (AVN), acute stress fractures, primary bone neoplasms (eg, osteoid osteoma), myeloproliferative bone marrow disorders (eg, leukemia), hemoglobinopathy (eg, sickle cell crisis), and infection (eg, septic and tuberculous osteomyelitis [1,2].

Painful conditions of the hip are often difficult to assess clinically, which may lead to a reliance on imaging for diagnosis. The diagnostic imaging pathway for hip pain has evolved considerably with the advent of MRI, supplanting bone scintigraphy as the investigation of choice for the assessment of symptomatic hip joint(s), in patients with acute non-traumatic hip pain whose radiographs are negative or inconclusive [3].

TOH, which is also termed transient BME, most commonly seen in middle-aged men and often occurring after trivial trauma or sport related injuries [4].

The current case report presents a female patient with TOH and extensive unilateral hip BME pattern that showed almost complete regression on follow-up MRI after 3 months of treatment with oral Alendronate Sodium 70 mg (Fosamax tablet, Merck research laboratories, United States). The case is discussed and the literature is reviewed regarding TOH and potential efficacy of Alendronate as off-label treatment in this domain.

## Case summary

**Fig. 1:**
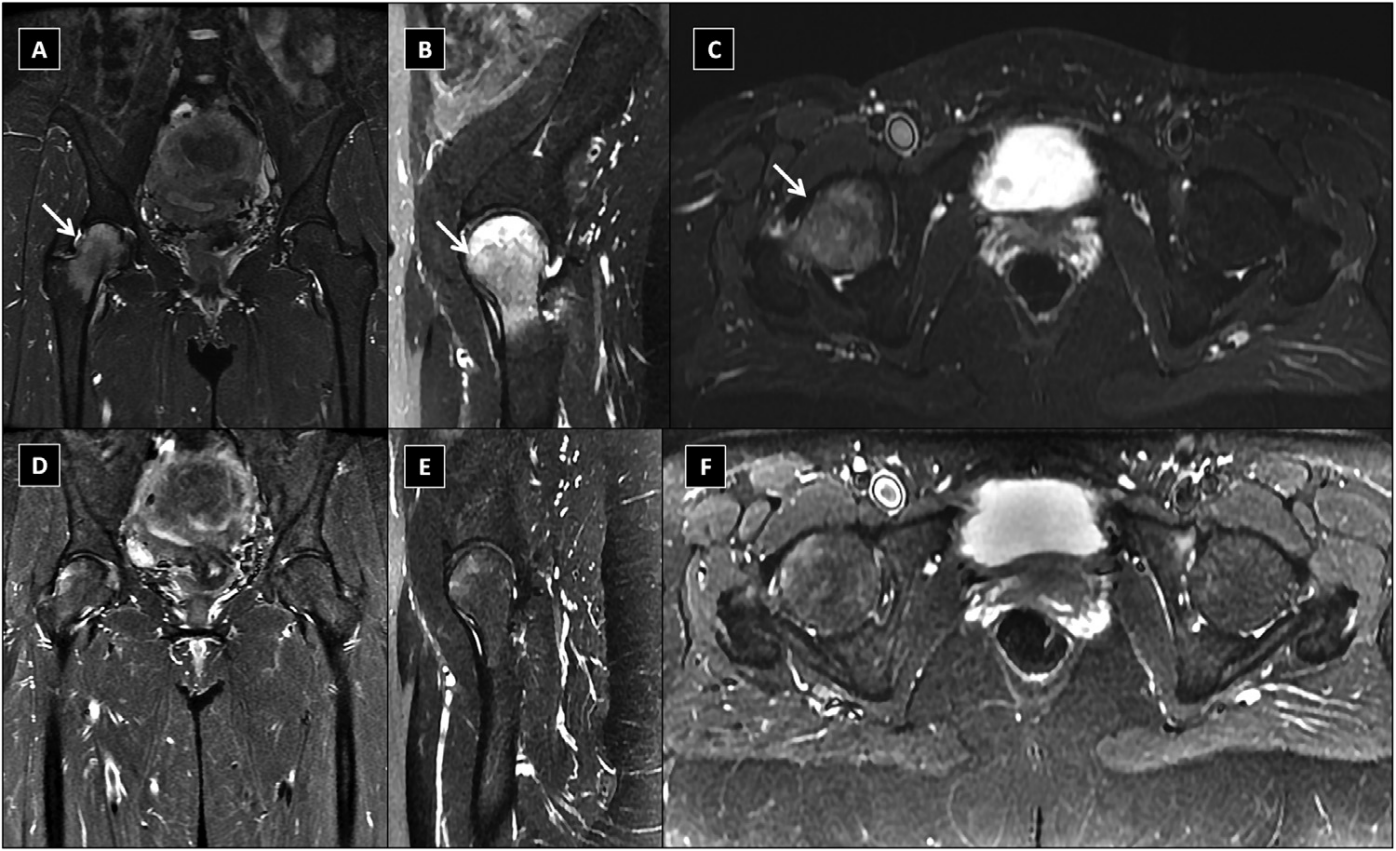
Initial magnetic resonance imaging (MRI) study for both hip joints: (a): coronal; (b) Sagittal; (c) axial Short tau inversion recovery (STIR) sequences: showing extensive bone marrow edema (BME) affecting the right femoral head, right femoral neck and extending to the intertrochanteric region: Follow-up MRI study showing the corresponding STIR sequences (d, e, –f) after 3 months of treatment with Alendronate showing almost total regression of the right femoral head neck marrow edema. The articular cartilages are preserved with no femoral head structural collapse could be detected.

A 39-year-old female patient presented with acute right knee pain and contusion after fall. During 2 months, she was treated for a right knee contusion, and an MRI of the right knee showed no signs of internal derangement. The patient started to develop progressive limping and presented in our facility for a second opinion. Careful clinical assessment indicated that the patient has a limping and antalgic gait pattern, as well as an inability to bear weight on the affected side and the right hip was painful on internal and external rotation. Further clinical assessment showed a positive Trendelenburg sign (with pelvis drops on the sound contralateral side (left) during a single leg stand on the affected right side). A radiograph of the hip joints showed no evidence of femoroacetabular impingement (FAI).Contrast enhanced MRI of both hip joints revealed extensive BME involving the right femoral head, right femoral neck and extending into the intertrochanteric region with no evidence of MRI signs suggesting AVN, (Figures 1 a, b, c) or any other pathology with no abnormal synovial enhancement on post contrast MRI. The patient was diagnosed as a case of unilateral TOH involving the right hip. She was prescribed oral Alendronate 70 mg weekly, as well as calcium and active vitamin-D Alfacalcidol1mcg (One-Alpha Capsules; Leo Laboratories Limited, Ballerup, Denmark)supplements, and she was strictly advised to avoid full weight bearing on the affected right hip. After 3 months of treatment, the patient continued to improve steadily, with considerable clinical improvement. Follow up MRI after three months of treatment showed almost total regression of the previously identified right femoral head-neck BME with preservation of the articular cartilage and no MRI signs of femoral head structural collapse could be identified (Figures 1 d–, e, f). The patient is advised to continue on the same lines of treatment for at least 6 months and to avoid full weight bearing on the affected limp. The salient facts in this report are that: TOH is a serious hip disorder with potential progression to femoral head collapse in case of missed or delayed diagnosis and oral Alendronate appears to be very effective line of treatment of TOH to prevent potential progression to AVN. The fact that the knee joint is innervated by branches of the femoral nerve anteriorly and branches of the sciatic, obturator, and saphenous nerves posteriorly explains why isolated knee pain can be the only symptom of hip diseases and is sometimes difficult to grasp.

## Discussion

TOH was first described by Curtiss and Kincaid in 1959 [5] as a syndrome of transient demineralization of the hip in the third trimester of pregnancy. In 1968, Lequesne was the first to use the term TOH in a published report [6]. There is no consensus regarding the management of TOH; in case of persistent BME condition it may progress to AVN of the involved hip. In a previous report Emad et al [4] described 8 patients with TOH who were successfully treated with oral Alendronate for 6 months resulting in improvement of hip pain and function with complete resolution of BME on follow-up MRI studies and none progressed to AVN [4]. The use of Alendronate to treat TOH in the study by Emad et al [4] was based on an findings reported by McCarthy [7] who described biopsy specimens from 19 cases with transient regional osteoporosis at different joints. The most important histopathological finding reported by McCarthy [7] is the presence of osteoclastic activity and bone in14 out of the 19 cases studied. Alendronate is chemically related to inorganic pyrophosphate, the latter inhibits osteoclastic activity and eventually inhibits bone resorption [8].

Furthermore, histological examination of core biopsy of the hip in patients with BME syndrome of the hip to assess bone metabolism revealed significantly raised levels of bone markers such as bone-specific alkaline phosphatase, osteocalcin, procollagen Type I N-terminal propeptide, and C-terminal cross-linking telopeptide, which were 4-16-fold greater than observed in serum. [9]. Moreover after progression to AVN, increased expression of angiogenesis factors (Vascular endothelial growth factor, Cysteine-rich 61, and connective tissue growth factor) has been observed in hip BME lesions. The higher levels were thought to imply a role for these proteins in osteonecrosis reparative processes [10].

Taken together, Alendronate efficacy in TOH is linked to inhibiting activated osteoclastic process and as a result preventing bone resorption that can lead to femoral head collapse and eventually AVN. In addition it has a potent analgesic effect on bone which is still an important issue in this domain [11].

In general BME syndromes (BMES) often refer to transient clinical conditions with unknown pathogenic mechanisms, including many entities such as TOH, regional migratory osteoporosis, and reflex sympathetic dystrophy. The disorder mainly affects the hip, the knee, and the ankle joints. Many hypotheses have been proposed to explain the pathogenesis of the disorder but unfortunately, the etiology of BMES remains obscure [12]. More recently in cam type of FAI, BME of the femoral head and neck was positively correlated with structural damage and synovitis, but not in pincer FAI type [13].

Some other Bisphosphonates showed similar efficacy in treating TOH [14,15]. Intravenous Pamidronate (45 mg was intravenously administered three times, once every third day), showed efficacy and MRI findings had normalized in 33 patients and bone mineral density values had improved. Furthermore intravenous Zoledronate showed similar efficacy in nine patients with TOH [15].

## Conclusions

TOH is a serious hip disorder with potential progression to AVN and femoral head collapse in case of missed or delayed diagnosis. Treatment with oral once weekly doses of Alendronate appears to be a very effective “off-label” line of treatment for TOH, with extensive BME pattern.

## Author statement

All the authors confirm that this work is never published before and currently not considered for publications elsewhere. All the authors have made substantial contributions to all of the following: (1) the conception and design of the report, acquisition of data, analysis and interpretation of data and drafting the article or revising it critically for important intellectual content and all approve this version to be submitted.

## Patient consent

The patient consent has been obtained.

## Declaration of Competing Interest

None to report.
